# A hypoxia- and telomerase-responsive oncolytic adenovirus expressing secretable trimeric TRAIL triggers tumour-specific apoptosis and promotes viral dispersion in TRAIL-resistant glioblastoma

**DOI:** 10.1038/s41598-018-19300-6

**Published:** 2018-01-23

**Authors:** Eonju Oh, JinWoo Hong, Oh-Joon Kwon, Chae-Ok Yun

**Affiliations:** 1 Department of Bioengineering, College of Engineering, Hanyang University, 222 Wangsimni-ro, Seongdong-gu, Seoul, 133-791 Korea; 2 Department of Molecular and Cellular Biology, Baylor College of Medicine, Houston, TX 77030 USA

## Abstract

Glioblastoma is a highly aggressive and malignant type of cancer that is apoptosis resistant and difficult to cure by conventional cancer therapies. In this regard, an oncolytic adenovirus that selectively targets the tumour tissue and induces tumour cell lysis is a promising treatment option. We designed and constructed a hypoxia-responsive and cancer-specific modified human telomerase reverse transcriptase (H5CmTERT) promoter to drive replication of an oncolytic adenovirus (H5CmTERT-Ad). To enhance the anti-tumour efficacy of H5CmTERT-Ad against malignant glioblastoma, we also generated an H5CmTERT-Ad expressing secretable trimeric tumour necrosis factor-related apoptosis-inducing ligand (H5CmTERT-Ad/TRAIL). H5CmTERT promoter-regulated oncolytic adenoviruses showed cancer-specific and superior cell-killing effect in contrast to a cognate control oncolytic adenovirus replicating under the control of the endogenous adenovirus promoter. The cancer cell-killing effects of H5CmTERT-Ad and H5CmTERT-Ad/TRAIL were markedly higher during hypoxia than normoxia owing to hypoxia responsiveness of the promoter. H5CmTERT-Ad/TRAIL showed more potent anti-tumour efficacy than H5CmTERT-Ad did in a xenograft model of TRAIL-resistant subcutaneous and orthotopic glioblastoma through superior induction of apoptosis and more extensive virus distribution in the tumour tissue. Altogether, our findings show that H5CmTERT-Ad/TRAIL can promote dispersion of an oncolytic adenovirus through robust induction of apoptosis in a highly TRAIL-resistant glioblastoma.

## Introduction

Glioblastoma is the most aggressive, invasive, and common form of human glioma. Despite decades of intensive research and advances in conventional anti-cancer modalities, patients with glioblastoma have the mean life expectancy of only 14.6 months^1,2^. Therefore, new therapeutic strategies for the effective treatment of glioblastoma are needed. Cancer gene therapy, which delivers a therapeutic gene into tumour cells, is a promising alternative to standard care^3^. To date, adenoviruses have been the most frequently utilized gene delivery vectors in clinical trials of gene therapy^4^. A cancer-specific and replication-competent adenovirus, i.e. an oncolytic adenovirus, is particularly promising for cancer gene therapy because the oncolytic adenovirus possesses intrinsic anti-tumour activity through replication-mediated lysis of cancer cells^5^. After cell lysis, amplified oncolytic adenovirus progenies are released and invade neighbouring cancer cells through secondary infection, ultimately resulting in a potent oncolytic effect due to lateral spread of the virus throughout the solid tumour^6–8^.

Utilization of a cancer cell-specific promoter has shown great potential for expressing exogenous genes in tumour tissues^9–12^. Prostate-specific antigen (PSA)-, α-fetoprotein (AFP)-, carcinoembryonic antigen (CEA)-, or other cancer type-specific promoters induce efficient therapeutic gene expression in a cancer cell-specific manner^9–12^. However, these cancer type-specific promoters can target only a single type of cancer expressing tumour antigens^13,14^. In stark contrast, the promoter of human telomerase reverse transcriptase (hTERT) is active in most types of tumours, namely, 90% of tumours strongly express telomerase, while its activity in healthy cells is minimal^15,16^. hTERT is the primary determinant of telomerase activity, and its activity can be increased by c-Myc- and Sp1-mediated regulation^17^. A modified hTERT promoter containing additional c-Myc- and Sp1-binding sites (mTERT) has been shown to induce stronger transcriptional activity than wild-type hTERT promoter can in tumour cells^18^, revealing that oncolytic adenoviruses replicating under the control of the mTERT promoter are good candidates for the treatment of cancers from various tissues of origin.

The tumour microenvironment is known to be hypoxic, having a median O_2_ level of 1.3%^19,20^. Hypoxia is a critical hurdle for the development of a successful treatment regimen against glioblastoma because hypoxia is known to make tumour cells more resistant to radio- and chemotherapy^21–23^. Furthermore, overexpression of hypoxia inducible factor (HIF)-1α during hypoxia promotes tumour growth^24^. Moreover, hypoxia attenuates viral replication of an oncolytic adenovirus in tumour tissue^25,26^. Therefore, a novel strategy is needed to deal with the hypoxic tumour microenvironment within a solid tumour and to improve the anti-tumour efficacy of oncolytic adenoviruses.

Malignant gliomas are known to be highly resistant to apoptosis, which is the main mechanism behind clinical benefits of radiation and chemotherapy^2^. Tumour necrosis factor (TNF)-related apoptosis-inducing ligand (TRAIL) is a strong therapeutic candidate for the treatment of glioblastoma because TRAIL can potently induce cancer-specific apoptosis^27^. Induction of TRAIL protein-mediated apoptosis in various types of tumour cells causes effective inhibition of tumour growth without substantial toxicity in various preclinical models^28–33^. The full-length TRAIL structural analysis by crystallography enabled the development of a secretable and trimeric form of TRAIL (amino acid residues 114–281; stTRAIL) containing a secretion signal, a trimerization domain, and an apoptosis-inducing domain of the TRAIL protein^34–36^. Secreted proteins, which circulate throughout the body, often have better access to target tissues than non-secreted proteins do, and this attribute is particularly important for therapeutic efficacy mediated by ligand molecules like TRAIL^37^.

In the present study, we generated a stTRAIL-expressing oncolytic adenovirus replicating under the control of a hypoxia-responsive and cancer-specific H5CmTERT promoter (H5CmTERT-Ad/TRAIL) to achieve robust and selective cancer cell-killing effect for the treatment of glioblastoma. We demonstrate that H5CmTERT-Ad/TRAIL can efficiently replicate and spread within a brain tumour, thus showing potent anti-tumour efficacy mediated by robust induction of apoptosis. Thus, H5CmTERT-Ad/TRAIL is a promising therapeutic option for the treatment of aggressive glioma.

## Results

### Construction of the H5CmTERT promoter and efficient stTRAIL expression by an H5CmTERT promoter-driven oncolytic adenovirus

The hTERT promoter is a good candidate for targeting of cancer because most cancers have some degree of telomerase activity^38,39^. Furthermore, several oncogenic transcription factors, such as c-Myc and HIF-1α, have been reported to transactivate the hTERT promoter^17^. We previously reported that an oncolytic adenovirus replicating under the control of mTERT containing an additional c-Myc-binding site and Sp1 sites has a potent and cancer-specific anti-tumour effect that is superior to that mediated by a wild-type hTERT promoter-driven oncolytic adenovirus^18^. In view of these findings, we inserted five additional c-Myc-binding sites upstream of the mTERT promoter, thus generating a 5CmTERT promoter to enhance the promoter transcriptional activity in cancer cells. 5CmTERT-Ad showed significantly higher cancer cell-killing efficacy than mTERT promoter-driven oncolytic adenovirus (mTERT-Ad) did, indicating that insertion of five additional c-Myc-binding sites greatly augmented transcriptional activity of the mTERT promoter (Supplementary Fig. 2b).

**Figure 1:**
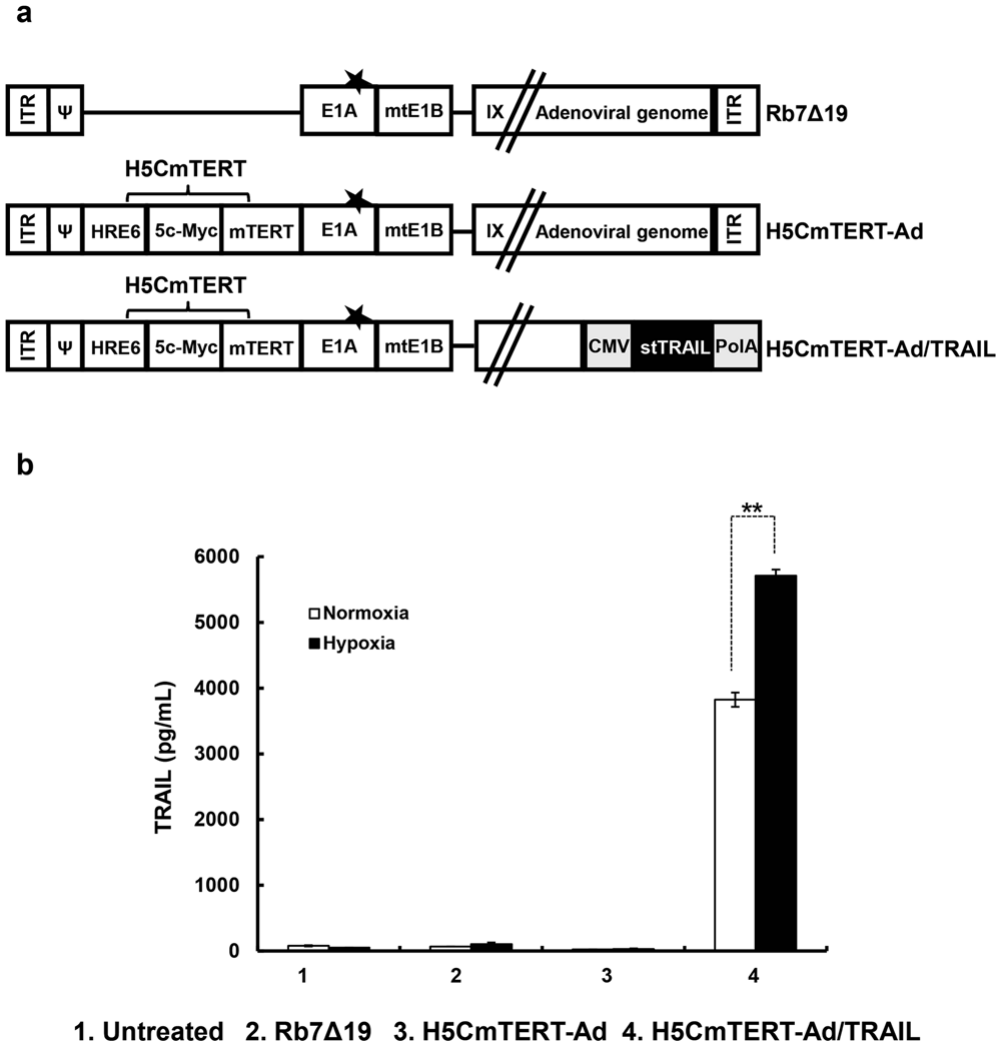
Construction and generation of H5CmTERT promoter-regulated oncolytic adenoviruses. (**a**) A schematic representation of the genomic structures of oncolytic adenoviruses used in this study. Rb7Δ19 contains retinoblastoma (Rb)-binding site-mutated E1A gene and E1B 55 kDa gene, but lacks E1B 19 kDa gene, and E1A expression is controlled by endogenous adenovirus promoter. H5CmTERT-Ad has a backbone similar to that of Rb7Δ19 but replicates under the control of the H5CmTERT promoter rather than the endogenous adenovirus promoter. H5CmTERT-Ad/TRAIL expresses stTRAIL from the adenovirus E3 region of the H5CmTERT-Ad backbone (★ indicates a mutation of the Rb-binding site of E1A). (**b**) U87MG human glioblastoma cells were infected with Rb7Δ19, H5CmTERT-Ad, or H5CmTERT-Ad/TRAIL at multiplicity of infection (MOI) of 0.5. The concentration of stTRAIL in the culture supernatant was measured by an ELISA. Data are presented as mean ± SD of triplicate experiments. **P < 0.01.

Another critical hurdle for an oncolytic adenovirus is the hypoxic tumour microenvironment, which attenuates viral replication and the spread of the oncolytic adenovirus^25,26^. To overcome this drawback, we inserted six copies of hypoxia response elements (HREs) upstream of the 5CmTERT promoter, thereby generating a hypoxia-responsive and cancer-specific H5CmTERT promoter. This promoter was utilized to drive replication of the H5CmTERT-Ad with the aim of enhancing the activity of the control oncolytic adenovirus (Rb7Δ19) replicating under the control of the endogenous adenovirus early region 1A (E1A) promoter, possesses mutated retinoblastoma (Rb) binding site in the adenovirus E1A gene, and deletion to E1B 19 kDa region under hypoxic conditions (Fig. 1a). To preferentially induce apoptosis of tumour cells and enhance therapeutic efficacy of the oncolytic adenovirus, we generated a H5CmTERT-Ad/TRAIL encoding the stTRAIL gene in the E3 region of the H5CmTERT-Ad backbone. As shown in Fig. 1b, H5CmTERT-Ad/TRAIL induced efficient expression of the stTRAIL gene during normoxia and hypoxia. Moreover, H5CmTERT-Ad/TRAIL yielded significantly higher stTRAIL expression during hypoxia (5712.0 ± 92.0 pg/mL) than normoxia (3824.0 ± 108.0 pg/mL; Fig. 1b; *P* < 0.01).

### The cancer-specific and potent cell-killing effect of H5CmTERT-Ad/TRAIL

**Figure 2:**
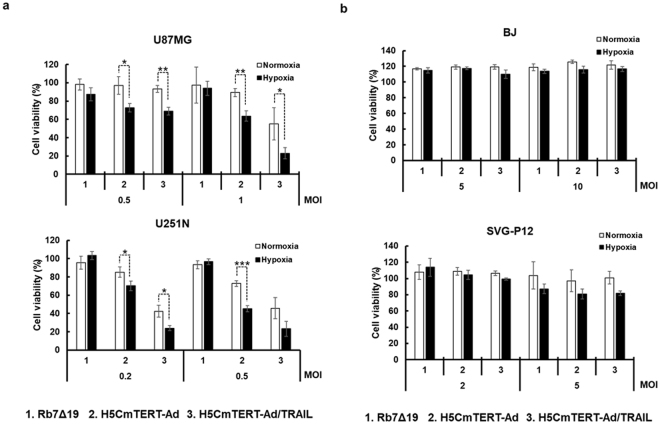
Cancer cell-specific killing effect of the oncolytic adenoviruses. (**a**) Glioblastoma cells (U87MG and U251N) and (**b**) normal cells (BJ and SVG-P12) were infected with Rb7Δ19, H5CmTERT-Ad, or H5CmTERT-Ad/TRAIL at the indicated  MOI and incubated under normoxic or hypoxic conditions. At 48 h after the infection, cell viability was measured by the 3-(4,5-dimethylthiazol-2-yl)-2,5-diphenyltetrazolium bromide (MTT) assay. Each cell line was tested at least three times, and data are shown as mean ± SD of triplicate experiments; *P < 0.05, **P < 0.01, ***P < 0.001.

To assess the cancer cell-killing effect and cancer specificity of the oncolytic adenoviruses during normoxia and hypoxia, a 3-(4,5-dimethylthiazol-2-yl)-2,5-diphenyltetrazolium bromide (MTT) assay was performed on glioblastoma cell lines (U87MG and U251N) and normal cell lines (BJ and SVG-P12) after infection of these cells with one of oncolytic adenoviruses at varying multiplicity of infection (MOI). As shown in Fig. 2a, H5CmTERT-Ad and H5CmTERT-Ad/TRAIL (*P* < 0.05, *P* < 0.01, or *P* < 0.001 versus Rb7Δ19 for U87MG, *P* < 0.01 or *P* < 0.001 versus Rb7Δ19 for U251N, respectively) had a dose-dependent cancer cell-killing effect that was superior to that of the Rb7Δ19 in glioblastoma cells. Furthermore, H5CmTERT-Ad/TRAIL elicited a more potent cancer cell-killing effect than H5CmTERT-Ad did during normoxia (*P* < 0.05 for U87MG, *P* < 0.05 for U251N, respectively) and hypoxia (*P* < 0.01 for U87MG, *P* < 0.05 for U251N, respectively). These data indicated that stTRAIL expression can augment the potency of the oncolytic adenovirus. At the highest viral dose examined, H5CmTERT-Ad/TRAIL elicited a 4.2- or 2.0-fold stronger cancer cell-killing effect than H5CmTERT-Ad did in glioblastoma cells (U87MG and U251N, respectively) during normoxia. Moreover, HRE-containing oncolytic adenoviruses (H5CmTERT-Ad and H5CmTERT-Ad/TRAIL) showed markedly higher cell killing efficacy during hypoxia than normoxia [normoxia vs. hypoxia; *P* < 0.05 or *P* < 0.01 for U87MG (0.5 MOI), *P* < 0.05 for U251N (0.2 MOI), respectively], indicating that HRE inserted upstream of a cancer-specific promoter can give hypoxia responsiveness to (and enhance the potency of) an oncolytic adenovirus. Of note, H5CmTERT-Ad and H5CmTERT-Ad/TRAIL were not cytotoxic toward normal cells (10- or 2-fold higher MOI was used for BJ and SVG-P12 than the highest dose used to treat glioblastoma cells, respectively), thus demonstrating good cancer specificity of the H5CmTERT-based oncolytic adenoviruses. These results also illustrate that the expression of stTRAIL did not harm normal cells, confirming that the stTRAIL-mediated cell-killing effect is indeed cancer specific. Taken together, these results indicate that the stTRAIL-expressing oncolytic adenovirus replicating under the control of the hypoxia-responsive and chimeric mTERT promoter had a potent and cancer-specific cytocidal effect under normoxic and hypoxic conditions.

### Potent induction of apoptotic cancer cell death by H5CmTERT-Ad/TRAIL

**Figure 3:**
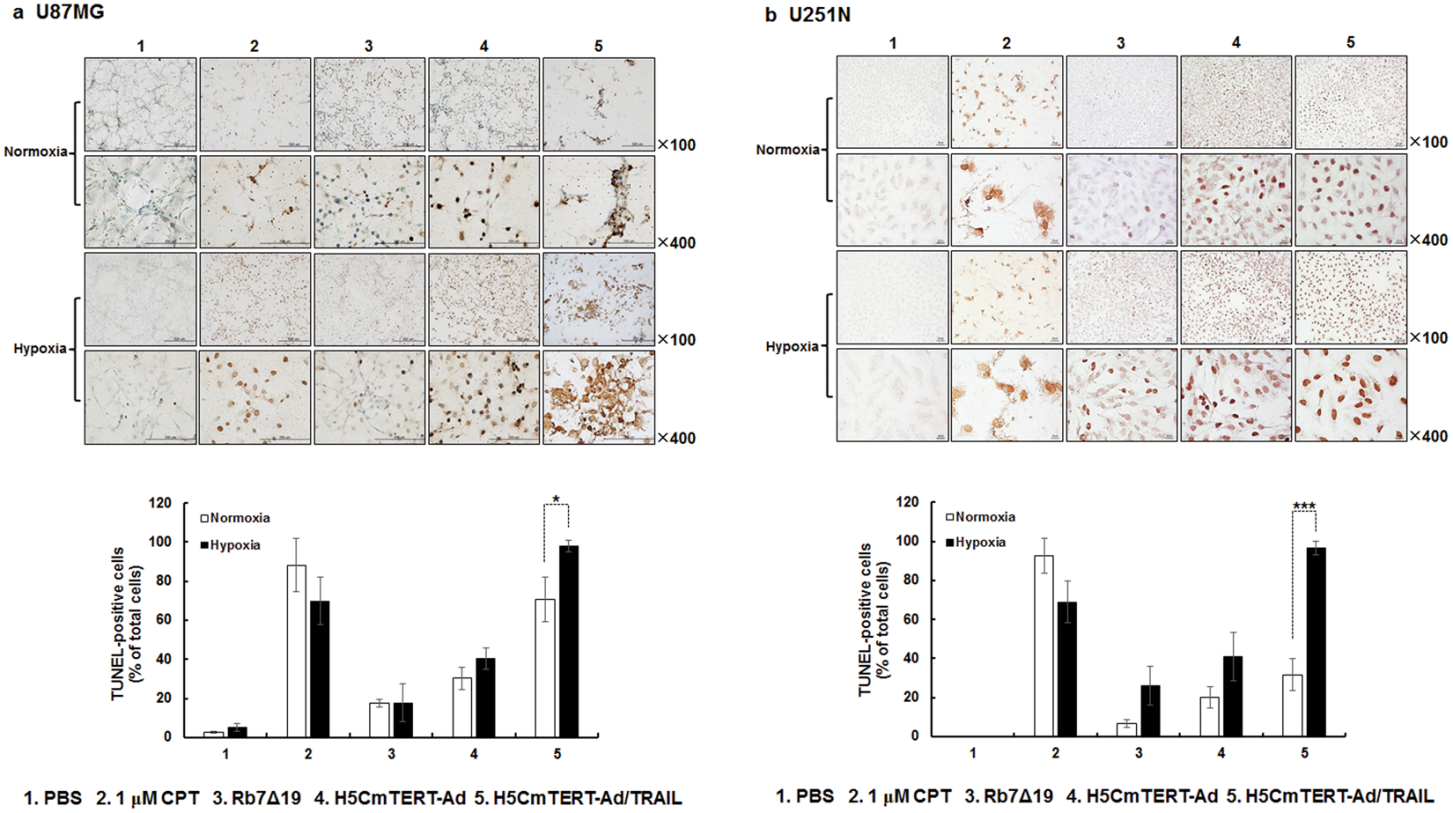
stTRAIL-mediated potent induction of apoptosis. (**a**) U87MG or (**b**) U251N cells were treated with 1 μM of camptothecin (CPT), Rb7Δ19, H5CmTERT-Rd19-Ad, or H5CmTERT-Ad/TRAIL. At 48 h post-treatment, a terminal deoxynucleotidyl transferase dUTP nick end labelling (TUNEL) assay was conducted. The cells were counted (TUNEL-positive vs total cell count) in five independent fields within microscopic images. The data are representative of three independent experiments conducted in triplicate; *P < 0.05, ***P < 0.001. CPT served as a positive control of induction of apoptosis. A representative field from three independent experiments is shown. Original magnification: ×100 and ×400.

To test whether the increased cell-killing effect of the stTRAIL-expressing oncolytic adenovirus was mediated by the induction of apoptosis, a terminal deoxynucleotidyl transferase dUTP nick end labelling (TUNEL) assay was performed. Camptothecin (CPT), a well-established chemical inducer of apoptosis, was used as a positive control for analysis of apoptotic cancer cell death. In both U87MG and U251N glioblastoma cells, H5CmTERT-Ad/TRAIL treatment yielded a markedly greater number of TUNEL-positive cells than Rb7Δ19 or H5CmTERT-Ad treatment did during normoxia, showing a 4.0- or 2.3-fold (U87MG) and 4.7- and 1.6-fold (U251N) higher percentage of apoptotic cells, respectively (Fig. 3a and b). Moreover, H5CmTERT-Ad/TRAIL-mediated induction of apoptosis was significantly enhanced under hypoxic conditions (normoxia vs. hypoxia; 70.8 ± 11.4% vs. 98.0 ± 2.8% for U87MG glioblastoma cells, 31.8 ± 8.4% vs. 96.4 ± 3.5% for U251N glioblastoma cells; *P* < 0.05 or *P* < 0.001). Altogether, these results indicate that oncolytic-adenovirus-mediated expression of stTRAIL can enhance the anti-cancer effect of an oncolytic adenovirus via induction of apoptosis.

### Morphological analysis of H5CmTERT-Ad/TRAIL-induced apoptosis in glioblastoma cells

**Figure 4:**
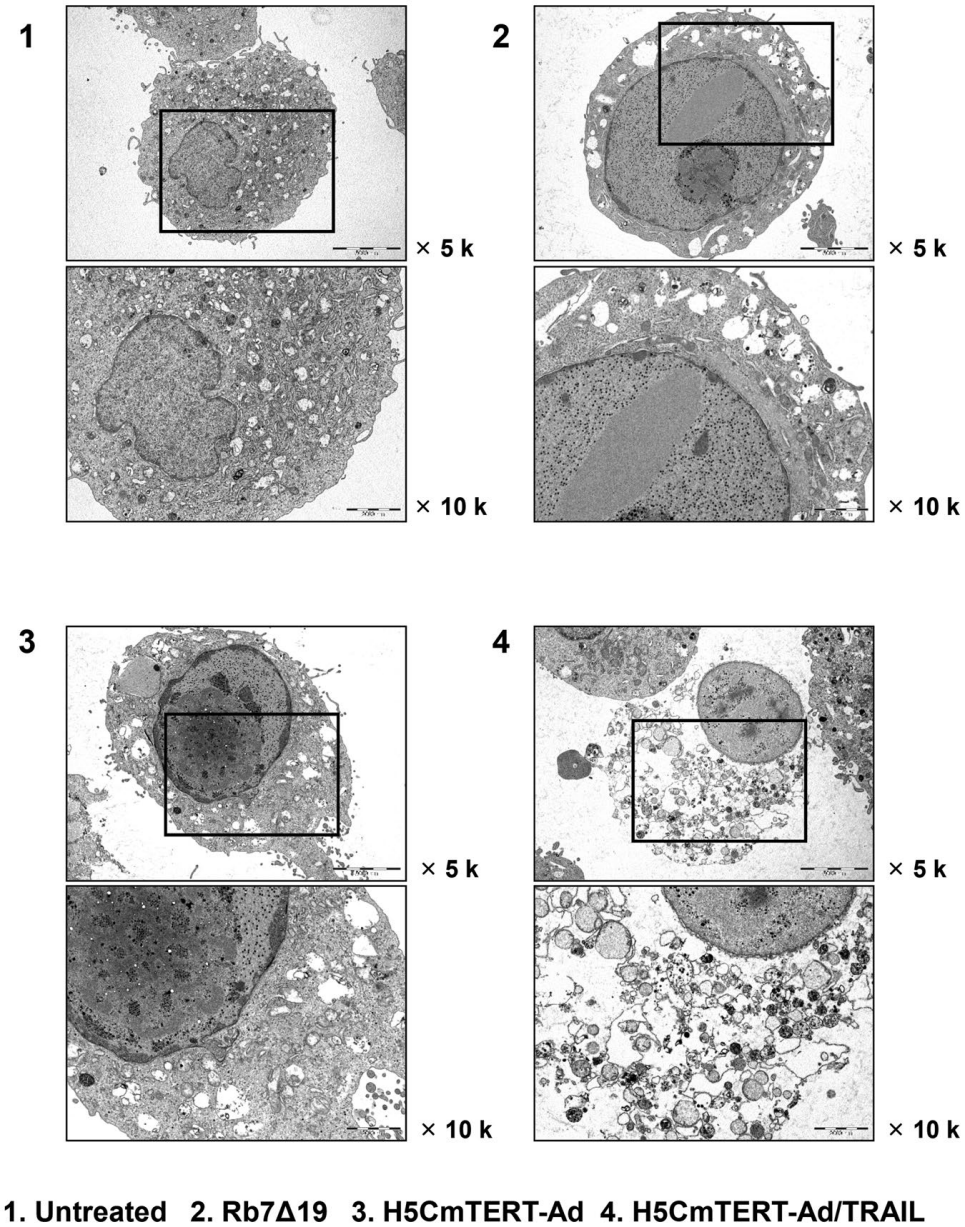
Transmission electron microscopy images of U87MG cells after infection with one of the oncolytic adenoviruses. U87MG cells were treated with PBS, Rb7Δ19, H5CmTERT-Ad, or H5CmTERT-Ad/TRAIL. At 36 h post-infection, the cells were harvested and analysed by transmission electron microscopy. Original magnification: ×5,000 and ×10,000.

To further assess the potent cancer cell killing mediated by H5CmTERT-Ad/TRAIL, we examined the morphology of U87MG glioblastoma cells treated with oncolytic adenoviruses by transmission electron microscopy. Rb7Δ19 or H5CmTERT-Ad-treated glioblastoma cells showed increased cell volume and nuclear space as compared to untreated cells, indicating that the oncolytic adenovirus induced necrosis of cancer cells^40^ (Fig. 4). Furthermore, all oncolytic-adenovirus-treated cells contained multiple adenovirus particles within the cytoplasm and nucleus. Oncolytic-adenovirus-infected cells showed condensation of chromatin into small, irregular, and circumscribed patches, indicating extensive cellular damage mediated by replication of the oncolytic adenovirus. Moreover, H5CmTERT-Ad/TRAIL-treated cells showed more extensive degradation of the plasma membrane and increased virus accumulation within cellular debris than did the cells treated with Rb7Δ19 or H5CmTERT-Ad, suggesting that stTRAIL expression can accelerate cellular degradation and facilitate the subsequent release of viral progenies.

### The potent anti-tumour effect of H5CmTERT-Ad/TRAIL against a glioblastoma  xenograft

**Figure 5:**
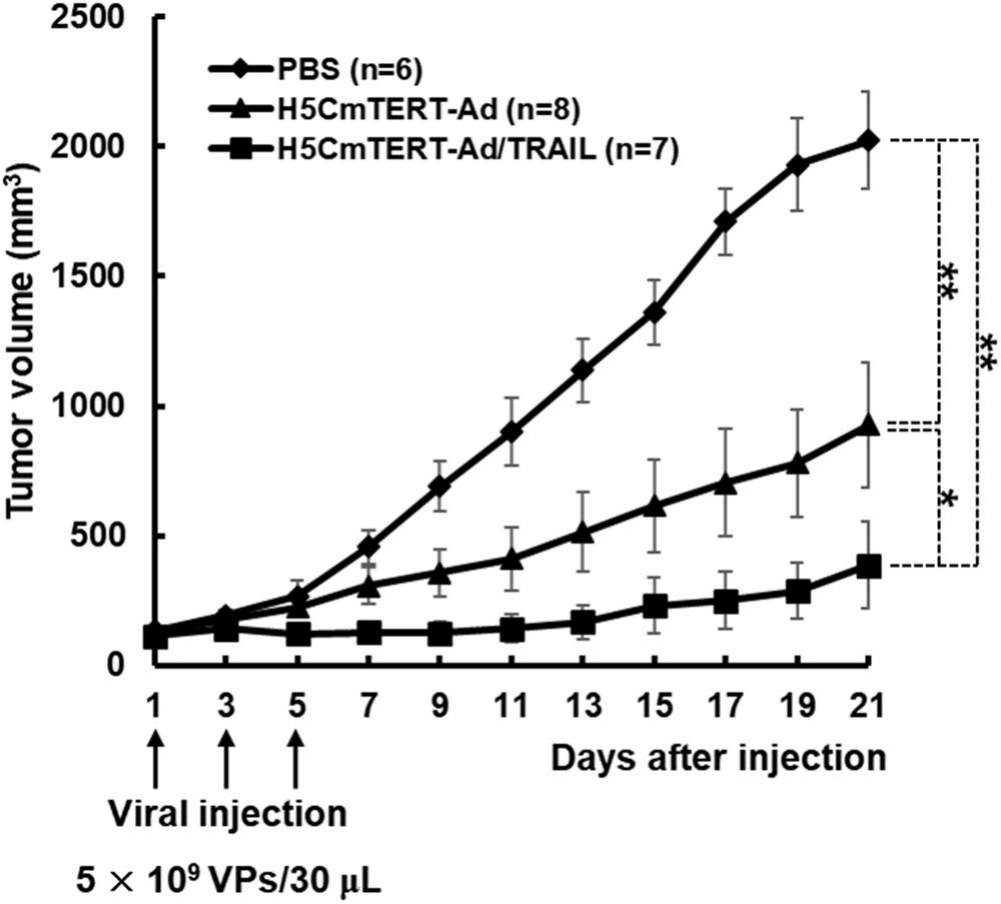
Assessment of the anti-tumour effect and survival rate in a subcutaneous glioblastoma xenograft model. U87MG glioblastoma xenograft tumours received three doses of PBS, H5CmTERT-Ad, or H5CmTERT-Ad/TRAIL (5 × 10^9^ VPs) via intratumoural injection on days 1, 3, and 5 (vertical arrows). Tumour growth was monitored at 2-day intervals by measuring the width (W) and length (L) of the tumour. Tumour volume was estimated with the following formula: volume = 0.523 × L × W^2^. *P < 0.05, **P < 0.01.

To evaluate the anti-tumour effect of H5CmTERT-Ad/TRAIL, the growth of established U87MG xenograft tumours was monitored following intratumoural injection of the phosphate-buffered saline (PBS), H5CmTERT-Ad, or H5CmTERT-Ad/TRAIL. As shown in Fig. 5, PBS-treated U87MG tumours increased up to an average size of 2,022.7 ± 186.0 mm^3^  at 21 days after the treatment. In contrast, H5CmTERT-Ad- or H5CmTERT-Ad-treated tumours reached an average size of 927.4 ± 240.6 and 385.4 ± 167.3 mm^3^, respectively, at 21 days, showing a 54.2% and 80.9% tumour growth inhibition compared with PBS (*P* < 0.01). It should be noted that H5CmTERT-Ad/TRAIL elicited 1.5-fold higher tumour growth inhibition than H5CmTERT-Ad did, indicating that oncolytic adenovirus-mediated stTRAIL expression in tumour tissue can enhance the anti-tumour efficacy of an oncolytic adenovirus (*P* < 0.05).

### Histological, immunohistochemical, and immunofluorescent analyses of the glioblastoma xenografts treated with H5CmTERT-Ad/TRAIL

**Figure 6:**
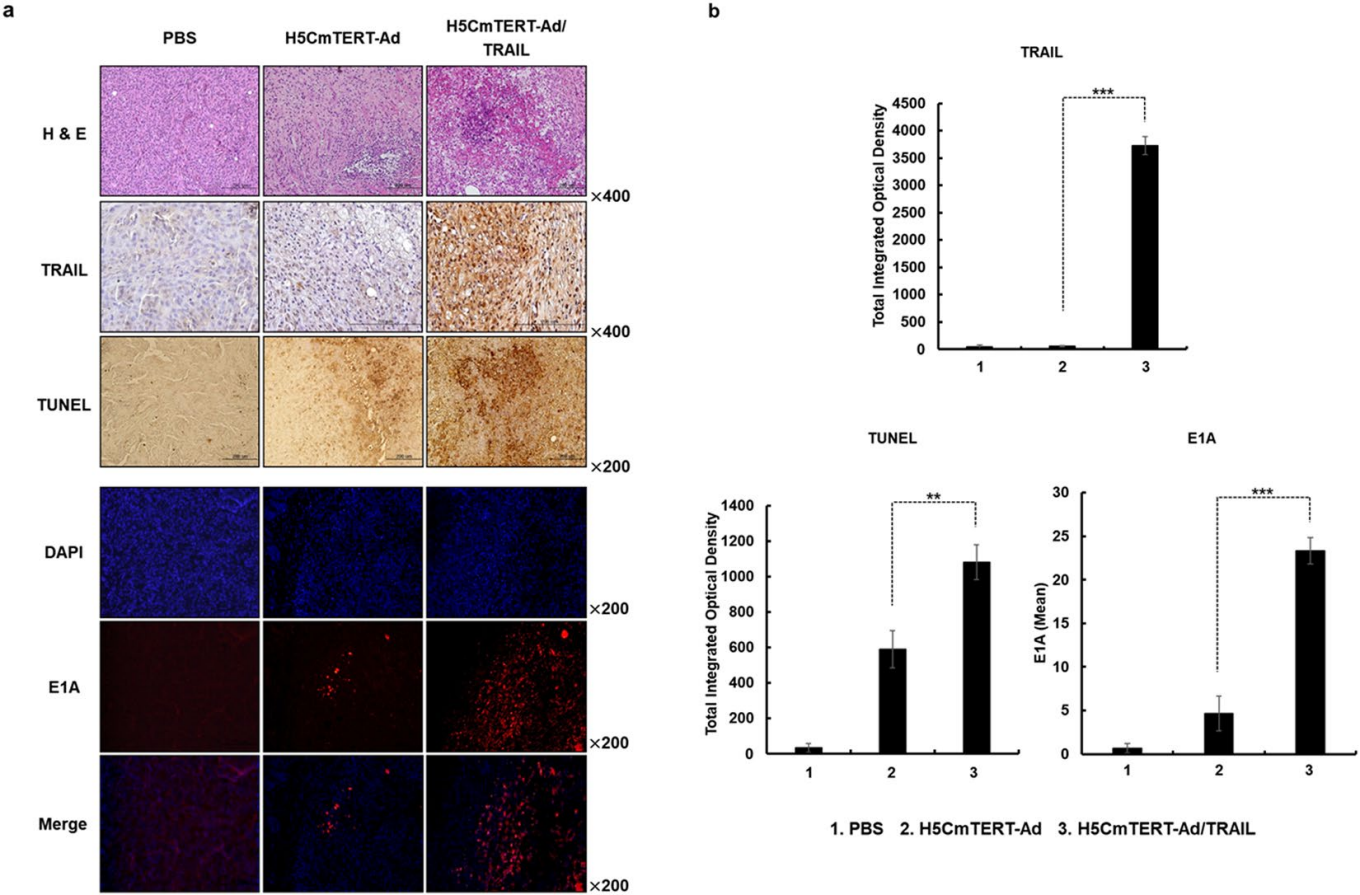
Histological and immunohistochemical analyses of tumour tissues treated with oncolytic adenoviruses. U87MG tumours established in nude mice were treated with PBS, H5CmTERT-Ad or H5CmTERT-Ad/TRAIL on days 1, 3, and 5, and the tumours were harvested on the 3^rd^ day after administration of the final treatment. (**a**) Representative sections were stained with hematoxylin and eosin (H & E). The expression levels of TRAIL and E1A were assessed by immunohistochemical analysis. A TUNEL assay was performed to detect apoptosis. Data are representative of three independent experiments. Original magnification: ×200 and ×400. (**b**) Semi-quantitative analysis of TRAIL-, TUNEL-, or E1A-stained sections in the MetaMorph image analysis software or ImageJ software. All the results are shown as means ± SD; **P < 0.01, ***P < 0.001.

To evaluate the mechanism underlying the potent anti-tumour effect of H5CmTERT-Ad/TRAIL, U87MG xenograft tumours were harvested on the 3^rd^ day after final treatment and subjected to histological, immunohistochemical, and immunofluorescent analyses. As shown in Fig. 6a, hematoxylin and eosin (H & E) staining revealed large areas of proliferating tumour cells in PBS-treated tissues, whereas markedly reduced numbers of tumour cells and moderate necrosis were observed in H5CmTERT-Ad- and H5CmTERT-Ad/TRAIL-treated tumour tissues. In particular, sections of H5CmTERT-Ad/TRAIL-treated tumours showed larger necrotic areas than did sections of tumours treated with H5CmTERT-Ad. Furthermore, H5CmTERT-Ad/TRAIL-treated tumours showed abundant stTRAIL expression, which led to stronger induction of apoptosis as compared with H5CmTERT-Ad (*P < *0.01 versus H5CmTERT-Ad), indicating that oncolytic-adenovirus-mediated expression of stTRAIL can enhance anti-tumour efficacy of an adenovirus by promoting apoptotic cell death (Fig. 6a and b). Moreover, H5CmTERT-Ad/TRAIL-treated tumours showed significantly greater quantity of viral progenies spread over larger areas than did those treated with H5CmTERT-Ad (*P < *0.001), suggesting that stTRAIL-induced apoptosis may facilitate secondary infection of neighbouring tumour cells and enhance the viral distribution of the oncolytic adenovirus in tumour tissue.

### Potent anti-tumour efficacy of intracranially administered H5CmTERT-Ad/TRAIL against an orthotopic glioblastoma xenograft

**Figure 7:**
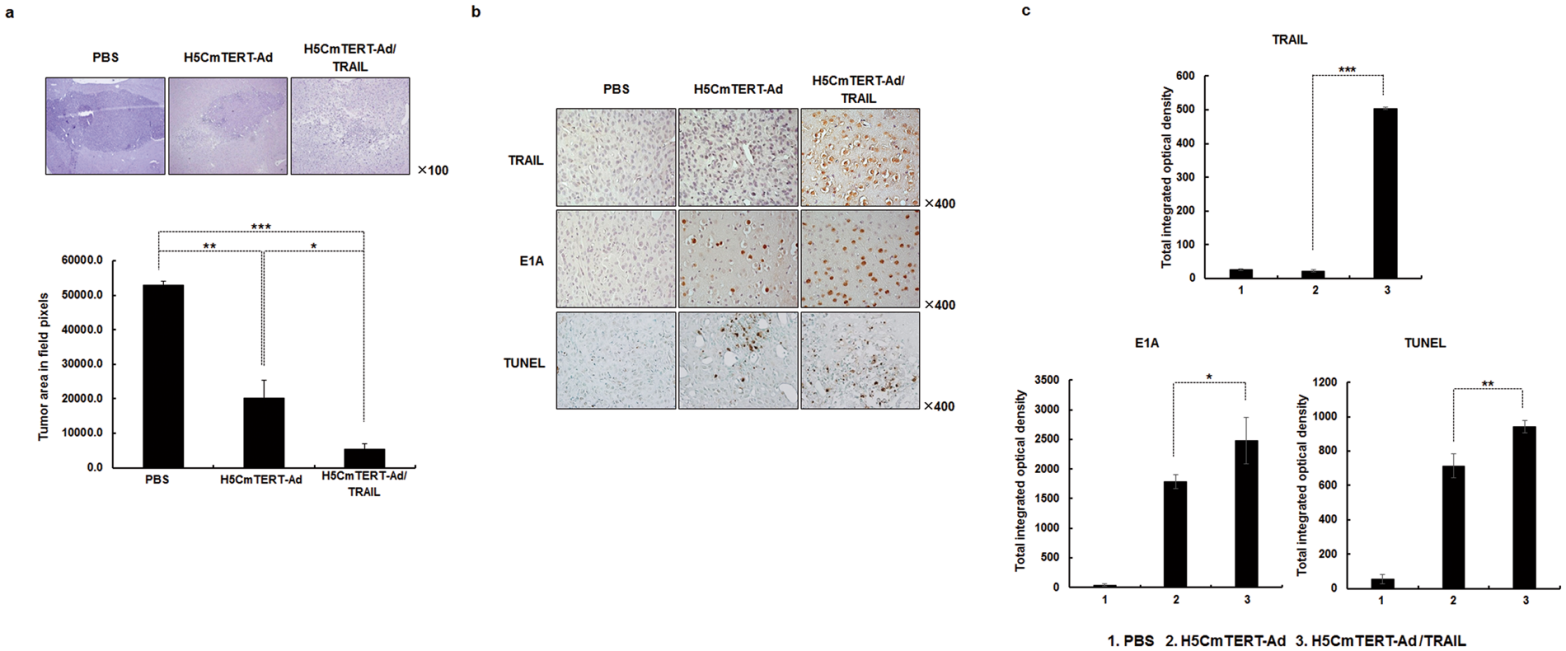
Anti-tumour effects of oncolytic adenoviruses in an orthotopic glioblastoma tumour model. Orthotopic brain tumour model was established by stereotaxically implanting U87MG cells into the left forebrain of nude mice. Seven days after tumour cell injection, PBS, H5CmTERT-Rd19-Ad, or H5CmTERT-Ad/TRAIL (5 × 10^9^ VPs) were administered via intracranial injection. (**a**) At 17 days after the cell injection, the mice were euthanised and the brains were collected. Representative tissue slices were stained with H & E to assess the tumour burden in each treatment group in the ImageJ software. Original magnification: ×100. *P < 0.05, **P < 0.01, ***P < 0.001. (**b**) The expression levels of TRAIL and E1A were assessed by immunohistochemistry. A TUNEL assay was carried out to detect apoptosis. Original magnification: ×400. (**c**) Semi-quantitative analysis of TRAIL-, E1A-, or TUNEL-stained sections using MetaMorph image analysis software. Images are representative of three independent experiments. All results are shown as mean ± SD; *P < 0.05, **P < 0.01, ***P < 0.001.

**Figure 8:**
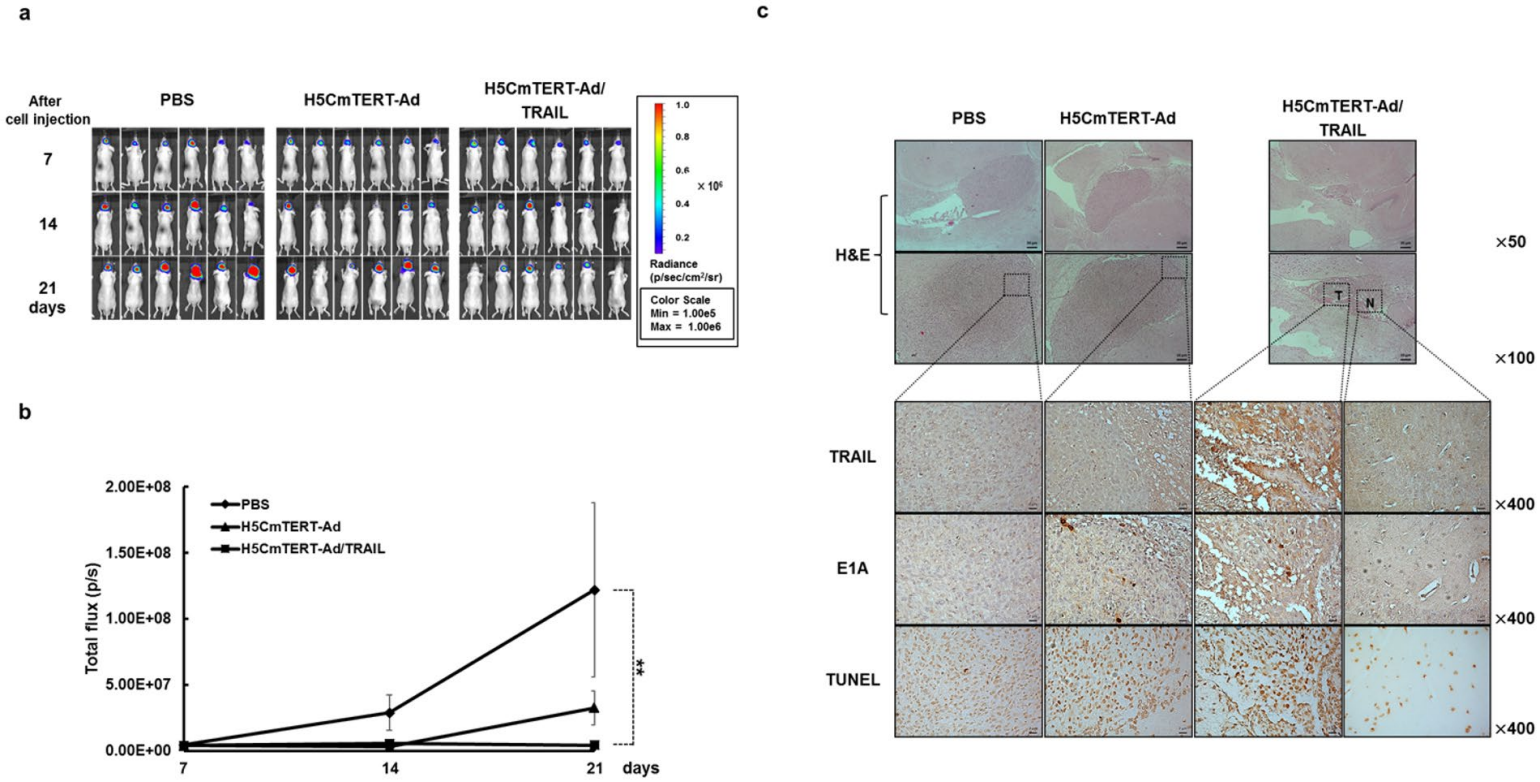
Anti-tumour effects of oncolytic adenoviruses in an U87MG/Fluc orthotopic glioblastoma tumour model. Orthotopic glioblastoma tumour model was established by stereotaxically implanting firefly luciferase-expressing U87MG (U87MG/Fluc) cells into the left forebrain of nude mice. At 7 days after the tumour cell injection, PBS, H5CmTERT-Rd19-Ad, or H5CmTERT-Ad/TRAIL (5 × 10^9^ VPs) were administered via intracranial injection. (**a**) Bioluminescence whole-body imaging was performed every 7 days following the treatments. (**b**) Bioluminescence signals were calculated after background subtraction in total flux photons/s from a body region of interest. Data presented as mean ± SD; **P < 0.01. (**c**) Histological and immunohistochemical analyses of tumour tissues from mice treated with PBS, H5CmTERT-Rd19-Ad, or H5CmTERT-Ad/TRAIL. At 10 days after the cell injection, the mice were euthanised and the brains were collected. Serially sectioned tumour tissues were stained with H & E. The expression levels of TRAIL and E1A were assessed by immunohistochemical analysis. A TUNEL assay was performed to detect apoptosis. Data are representative of three independent experiments. Original magnification: ×50, ×100, and ×400 magnification of the boxed area. T, tumour tissues; N, normal tissues in the H5CmTERT-Ad/TRAIL-treated group.

The orthotopic tumour models are reliable *in vivo* preclinical models for testing new drugs and therapies because of their close resemblance of clinical cancer^41^. To determine whether H5CmTERT-Ad/TRAIL can suppress the growth of glioblastoma in its natural niche, orthotopic glioblastoma xenografts were established by stereotaxically injecting U87MG glioblastoma cells into the left forebrain of athymic nude mice. On the 7^th^ day after cell injection, the mice were intracranially injected with PBS, H5CmTERT-Ad, or H5CmTERT-Ad/TRAIL. As shown in Fig. 7a, the average area of a tumour treated with PBS reached 53,001.7 ± 1,142.9 pixels by the 17^th^ day after the cell injection. In contrast, mice treated with an oncolytic adenovirus (H5CmTERT-Ad or H5CmTERT-Ad/TRAIL) had a significantly reduced tumour area in comparison with those treated with PBS; H5CmTERT-Ad and H5CmTERT-Ad/TRAIL caused 61.7% and 89.8% suppression, respectively (*P* < 0.01 for H5CmTERT-Ad, *P* < 0.001 for H5CmTERT-Ad/TRAIL versus PBS). It should be noted that H5CmTERT-Ad-treated tumour tissues showed a 73.4% smaller tumour region than did those treated with H5CmTERT-Ad (*P* < 0.05), indicating that stTRAIL expression by H5CmTERT-Ad/TRAIL can enhance the anti-tumour effect of the oncolytic adenovirus against orthotopic glioblastoma. Importantly, H5CmTERT-Ad/TRAIL induced potent tumour growth inhibition up to 21 days post cell injection in U87MG/Fluc orthotopic glioblastoma tumour model (*P* < 0.01 for H5CmTERT-Ad/TRAIL versus PBS) (Fig. 8a and b), showing similar results as those observed in Figs 5 and 7. Furthermore, both H5CmTERT-Ad- and H5CmTERT-Ad/TRAIL-treated mice showed higher long-term survival rate than those treated with PBS (Supplementary Fig. 6; *P* < 0.05 and *P* < 0.01, respectively). Importantly, H5CmTERT-Ad/TRAIL-treated mice showed 7.5-fold higher tumour growth inhibition than H5CmTERT-Ad-traeted mice at 21 days post cell injection. Furthermore, 33.3% of H5CmTERT-Ad/TRAIL-treated mice were viable at 35 days post cell injection, whereas only 16.7% of H5CmTERT-Ad-treated mice were viable in the same time period. Together, these results suggest that H5CmTERT promoter-regulated oncolytic Ad can effectively suppress the growth of aggressive orthotopic glioblastoma tumours through expression of *TRAIL* gene.

To verify the mechanism underlying the improved therapeutic efficacy of H5CmTERT-Ad/TRAIL, U87MG orthotopic tumours were harvested 17 days after cell injection and then evaluated by immunohistochemical analysis. A large amount of stTRAIL was observed in the tumour tissues treated with H5CmTERT-Ad/TRAIL (Fig. 7b and c). In addition, H5CmTERT-Ad/TRAIL-treated tumour tissues showed significantly greater accumulation of the oncolytic adenovirus and a greater apoptotic tumour cell population as compared to those treated with H5CmTERT-Ad (*P* < 0.05 and *P* < 0.01, respectively). Additionally, immunohistochemical analysis of serially sectioned U87MG/Fluc-harboring brain tissues was performed. In line with our results from Fig. 7a–c, H & E  staining revealed that H5CmTERT-Ad/TRAIL-treated mice had lower tumour burden and markedly higher accumulation of oncolytic adenovirus than H5CmTERT-Ad-treated mice (Fig. 8c). Furthermore, H5CmTERT-Ad/TRAIL-treated tumours showed abundant stTRAIL expression and strong induction of apoptosis in the serially sectioned tumour tissues, whereas no stTRAIL- and minimal TUNEL-positive spots were observed in surrounding normal tissue. Of note, the low quantity of TUNEL-positive spots observed in normal brain region following H5CmTERT-Ad/TRAIL was observed at a similar level in normal brain tissues without any treatment (Supplementary Fig. 5). When H5CmTERT-Ad/TRAIL was directly injected into normal brain tissues of healthy mice, it did not replicate, express TRAIL, nor induce any additional apoptosis. Together, these findings suggest that H5CmTERT-Ad/TRAIL can effectively replicate, express TRAIL, and induce apoptosis in a highly tumour-restricted manner to elicit potent anti-tumour efficacy against orthotopic glioblastoma tumour.

## Discussion

Malignant gliomas remain devastating primary brain tumours and such patients have a poor survival rate. Glioblastoma is characterized by uncontrolled cellular proliferation, propensity for recurrence, and resistance to apoptosis; these properties severely restrict the efficacy of standard treatments. In this regard, cancer gene therapy has emerged as a promising approach to treatment of glioblastoma^1,42^. However, the results of initial gene therapy trials in glioblastoma have been disappointing due to limited therapeutic gene transfer efficiency and insufficient cancer specificity^43^.

To overcome these limitations, we generated a chimeric hTERT promoter (H5CmTERT) containing multiple copies of HREs and several additional c-Myc-binding sites to drive the replication of an oncolytic adenovirus (Fig. 1a). Under normoxic and hypoxic conditions, the two H5CmTERT promoter-driven oncolytic adenoviruses (H5CmTERT-Ad and H5CmTERT-Ad/TRAIL) elicited highly cancer-specific and more potent glioblastoma cell-killing effect than Rb7Δ19 did, which expresses an adenovirus gene under control of the endogenous adenovirus promoter (Fig. 2). The potent anti-cancer efficacy of H5CmTERT-Ad-driven oncolytic adenoviruses could be attributed to the following factors: (1) insertion of additional c-Myc-binding sites enhanced the transcriptional activity in glioblastoma cells because c-Myc proto-oncogene expression has been found to positively correlate with the grade of malignancy in glioblastoma^44^ and (2) insertion of HREs augmented the promoter activity under hypoxic conditions^45–47^. Of note, HRE-mediated enhancement of transcriptional activity of the H5CmTERT promoter did not affect cancer selectivity of the oncolytic adenovirus because both versions of H5CmTERT-Ad killed normal cells negligibly under normoxic and hypoxic conditions. These data are in good agreement with another report showing that the addition of HREs upstream of a cancer-specific promoter does not impair this promoter’s cancer specificity under hypoxic conditions^48^.

Malignant glioblastomas are known to be highly resistant to the proapoptotic effect of standard cancer therapy owing to constitutive activation of the phosphoinositide 3-kinase (PI3K)/Akt^49^, mammalian target of rapamycin (mTOR)^50^, or nuclear factor (NF)-κB^51–53^ signalling pathway^54,55^. It is noteworthy that the strong transcriptional activity of the H5CmTERT promoter enabled H5CmTERT-Ad/TRAIL to express stTRAIL abundantly and led to effective induction of apoptosis in TRAIL-resistant U87MG^31^ and TRAIL-sensitive U251N^56,57^ glioblastoma cells (Figs 1b and 3), suggesting that highly localized expression of stTRAIL in tumour tissue by an oncolytic adenovirus may overcome apoptosis resistance of malignant glioblastomas (Fig. 6). These findings are in line with other reports suggesting that localized expression of stTRAIL can induce more robust apoptosis than the expression of full-length TRAIL and that adenovirus-mediated expression of TRAIL can overcome TRAIL resistance^27,58^. Furthermore, H5mTERT-Ad/TRAIL was not noticeably cytotoxic toward normal cells, as was the case for control H5CmTERT-Ad, suggesting that stTRAIL-mediated apoptotic cell death is cancer specific (Fig. 2). Similar results were observed *in vivo*; H5mTERT-Ad/TRAIL exhibited more potent anti-tumour effect than its control H5CmTERT-Ad via stTRAIL-mediated induction of apoptosis and active viral replication within highly aggressive orthotopic glioblastoma tumour tissues. Moreover, the normal brain tissue adjacent to the tumour was not affected by H5mTERT-Ad/TRAIL (Fig. 8c and Supplementary Fig. 5). These finding are in good agreement with several reports showing that TRAIL selectively induces apoptosis in transformed cells and cancer cells but not in most normal cells^2,58–60^.

Similar trends were observed in both subcutaneous and orthotopic xenograft models of glioblastoma. In particular, H5CmTERT-Ad/TRAIL induced more potent inhibition of tumour growth than control H5CmTERT-Ad did, indicating that oncolytic-adenovirus-mediated expression of stTRAIL can enhance the anti-tumour efficacy of an adenovirus (Fig. 5). In support of these findings, a strong positive correlation between stTRAIL expression and apoptotic cell death was observed (Fig. 6a and b), suggesting that exogenous expression of stTRAIL can effectively induce apoptosis of glioblastoma *in vivo*. Of note, the magnitude of apoptosis and viral distribution also showed a strong positive correlation in the glioblastoma xenograft model: H5CmTERT-Ad/TRAIL (with a higher magnitude of apoptosis) also yielded more extensive viral distribution within tumour tissue than H5CmTERT-Ad did. This phenomenon can be mediated by two distinct mechanisms: (1) stTRAIL expression increases formation of apoptotic bodies, and these apoptotic bodies transfer viral progenies to neighbouring cancer cells^61^, and (2) extensive apoptotic cell death can generate a void and decrease the interstitial pressure within a solid tumour, ultimately facilitating lateral spread of the virus through enhanced diffusion^62^. In good agreement with these reports, H5CmTERT-Ad/TRAIL-mediated apoptotic cell death was associated with large numbers of virions in cellular debris, suggesting that the oncolytic adenovirus may be transferred to neighbouring tumour cells by apoptotic bodies **(**Supplementary Fig. 4). Moreover, similarly potent anti-tumour efficacy was observed in the orthotopic glioblastoma model (Figs 7 and 8). However, *in vitro* therapeutic effect by H5CmTERT-Ad/TRAIL showed more potent anticancer effect than *in vivo*. These discrepancy is due to complex nature of tumour microenvironment, which encompasses factors such as dense network of blood vessels, immune cells, stromal cell, and extracellular matrix, functioning as a critical limiting factor toward therapeutics^63^. In addition, H5CmTERT-Ad/TRAIL-mediated improvement in the survival of glioma-bearing mice, in respect to control H5CmTERT-Ad-treated mice, was less obvious in an orthotopic tumour model than subcutaneous tumour model. This is likely due to orthotopic tumours, which grow under an environmental condition that closely recapitulates their natural habitat, exhibiting more aggressive tendencies than subcutaneous tumours^64–68^.

In conclusion, hypoxia-responsive and highly cancer-specific H5CmTERT promoter-driven oncolytic adenoviruses can efficiently replicate under normoxic or hypoxic conditions and thus have a potent glioblastoma cell-killing effect. This potent anti-cancer effect of the H5CmTERT promoter-driven oncolytic adenovirus is further enhanced by expression of stTRAIL, which greatly enhances induction of apoptosis and viral distribution in both subcutaneous and orthotopic xenograft models of glioblastoma that are resistant to TRAIL-mediated apoptosis. Taken together, these attributes make H5CmTERT-Ad/TRAIL a promising candidate for a future clinical trial for treatment of glioblastoma.

## Materials and Methods

### Cell lines and cell culture

A human embryonic kidney cell line expressing the adenovirus E1 region (HEK293), glioblastoma cell lines (U87MG and U251N), and normal cell lines (BJ and SVG-P12) were purchased from the American Type Culture Collection (ATCC, Manassas, VA). All the cell lines were cultured in Dulbecco’s modified Eagle’s medium (DMEM; Gibco BRL, Grand Island, NY) supplemented with 10% of foetal bovine serum (FBS; Gibco BRL), 2 mM L-glutamine (Gibco BRL), and 100 IU/mL penicillin-streptomycin (Gibco BRL). All the cell lines were maintained at 37 °C in a humidified atmosphere containing 5% of CO_2._ Hypoxia was applied to the cells by means of prewarmed aluminium hypoxic chambers, and the oxygen concentration was maintained at 1%.

### Animal studies

Six-week-old athymic nude mice (Orientbio, Seongnam, Korea) were maintained in a laminar air-flow cabinet with specific pathogen-free conditions. All facilities were approved by the Association for Assessment and Accreditation of Laboratory Animal Care. All animal studies were performed according to the institutionally approved protocols of Hanyang University.

### Construction and generation of an H5CmTERT promoter-regulated oncolytic adenovirus

To generate an oncolytic adenovirus, a vector based on human adenovirus serotype 5, adenovirus E1A gene was placed under the control of the H5CmTERT promoter. We used the adenovirus E1 shuttle vector containing mutated a Rb-binding site in the adenovirus *E1A* gene and deletion in the E1B 19-kDa region (pDE1sp1B/Rb7Δ19)^69^ as a template plasmid. First, the H5CmTERT promoter was inserted into pDE1sp1B/Rb7Δ19, resulting in the pDE1sp1B/H5CmTERT/Rb7Δ19 adenovirus E1 shuttle vector. For homologous recombination with the linearized adenovirus dE1, the *Xmn*I-treated pΔE1sp1B/H5CmTERT/Rb7Δ19 adenovirus E1 shuttle vector was co-transfected into *Escherichia coli* BJ5183, generating pH5CmTERT-Ad. The proper recombinant adenovirus DNA was digested with *Pac*I and transfected into HEK293 cells to generate an H5CmTERT-Ad. The propagation, purification, and titration of all the adenoviruses used in this study were performed as previously described^70^. The number of viral particles (VPs) was calculated from optical density measurement at 260 nm (OD_260_), where absorbance of 1.0 (OD_260_ = 1) was equivalent to 1.1 × 10^12^ VP/mL. Purified viruses were stored at −80 °C until use.

### Construction and generation of a stTRAIL-expressing adenovirus

To generate an H5CmTERT promoter-regulated oncolytic adenovirus expressing stTRAIL in the adenovirus E3 region, we first constructed a pSP72-E3 adenovirus E3 shuttle vector expressing stTRAIL. The *stTRAIL* gene excised from pGT2SEC(CV)ILZTRAIL^37^ was first subcloned into the adenovirus E3 shuttle vector using *Bgl*II, creating the pSP72-E3/TRAIL E3 shuttle vector. The newly constructed pSP72-E3/TRAIL E3 shuttle vector was then linearized with *Xmn*I digestion, and H5CmTERT-Ad was linearized with *Spe*I digestion. The linearized pSP72-E3/TRAIL E3 shuttle vector was then co-transfected into *E. coli* BJ5183 along with the *Spe*I-digested pH5CmTERT-Ad for homologous recombination, resulting in the pH5CmTERT-Ad/TRAIL adenovirus vector. Lastly, HEK293A cells were transfected with viral DNA to generate H5CmTERT-Ad/TRAIL as described above.

### An enzyme-linked immunosorbent assay (ELISA) of stTRAIL expression

U87MG cells (7 × 10^8^) were plated in a 100-mm^3^ culture dish and then infected with one of oncolytic adenoviruses (Rb7Δ19, H5CmTERT-Ad, or H5CmTERT-Ad/TRAIL) at MOI of 0.5. To remove pre-existing human TRAIL from the medium, cells in each well were washed three times with 3 mL of the serum-free medium 30 h prior to harvesting and were incubated in a serum-free medium for additional 6 h. The cells were then further incubated for 24 h in the DMEM medium containing 5% of FBS. TRAIL expression was determined using a human TRAIL ELISA kit (BD Pharmingen, San Diego, CA).

### MTT assay

To evaluate the cancer cell-killing efficacy of the oncolytic adenoviruses, U87MG, U251N, BJ, and SVG-P12 cells grown to 60% confluence in 96-well plates were infected with Rb7Δ19, H5CmTERT-Ad, or H5CmTERT-Ad/TRAIL at various MOIs. At 2 days post-infection, 200 μL of MTT (Sigma-Aldrich, St. Louis, MO) in PBS (2 mg/mL) was added into each well. After 4 h incubation at 37 °C, the supernatant was discarded and the precipitate was dissolved in 200 μL of dimethyl sulfoxide (DMSO). The plates were then analysed on a microplate reader at 540 nm. Absorbance recorded in uninfected cells was assumed to represent 100% cell viability.

### Detection of apoptosis by the TUNEL assay *in vitro*

U87MG and U251N cells were plated on coverslips and treated with 1 μM CPT, Rb7Δ19, H5CmTERT-Ad, or H5CmTERT-Ad/TRAIL at MOI of 1.0. After 48 h, the TUNEL assay was carried out according to the manufacturer’s protocol (Merck, Darmstadt, Germany). The cells were counted (TUNEL-positive vs. total cell count) in five randomly selected high-power fields (original magnification: ×400).

### Transmission electron microscopy

U87MG cells grown to 80% confluence in a 60-mm^3^ dish were infected with Rb7Δ19, H5CmTERT-Ad, or H5CmTERT-Ad/TRAIL at MOI 1.0. After 36 h, the cells were harvested and prepared as described elsewhere^71^.

### Assessment of the anti-tumour effect in a subcutaneous glioblastoma xenograft model

To assess the anti-tumour effect of oncolytic adenoviruses, U87MG xenografts were subcutaneously established by injecting 10^7^ cells into the abdomen of 6-week-old male athymic nude mice (Orientbio) (n = 6, 8, or 7). When tumours reached an average volume of 100–150 mm^3^, the mice were intratumourally injected with PBS, H5CmTERT-Ad, or H5CmTERT-Ad/TRAIL (5 × 10^9^ VPs) on days 1, 3, and 5. The first day of treatment was designated as day 1. Tumour growth was monitored every other day by measuring the length (*L*) and width (*W*) of each tumour with a caliper, and tumour volume was calculated according to the formula: tumour volume = 0.523 × *L* × *W*^2^.

### Histological, immunohistochemical, and immunofluorescence evaluation of subcutaneous glioblastoma tumour tissue

Tumour tissues were collected from mice 3 days after the final treatment. Tumour tissues were fixed in 4% formalin and embedded in paraffin. Representative tissue slides were stained with H & E. Tumour slides were also stained with a mouse anti-TRAIL antibody (Ab) (BD PharMingen) to assess the expression of stTRAIL in tumour tissue. After overnight incubation with primary Abs at 4 °C, tumour slides were processed with an ABC-peroxidase kit (ChemoMate DAKO Envision kit; DAKO, Carpinteria, CA). To assess viral replication in tumour tissues by immunofluorescence analysis, the slides were stained with a rabbit anti-E1A IgG Ab (Santa Cruz Biotechnology, Santa Cruz, CA) and then incubated with an Alexa Fluor 568-conjugated anti-mouse IgG (secondary) Ab (Invitrogen, Carlsbad, CA). Nuclear staining with 4′,6-diamidino-2-phenylindole (DAPI, Sigma-Aldrich) was also performed. The induction of apoptosis (TUNEL-positive spots) in tumour tissue was assessed by means of a TUNEL assay kit (Merck). All slides were counterstained with Mayer’s hematoxylin. The expression levels of stTRAIL and TUNEL-positive cells (apoptotic cells) were semi-quantitatively analysed using the MetaMorph image analysis software (Universal Image, Westchester, PA). E1A expression was semi-quantitatively analysed in the ImageJ software (version 1.50b; U.S. National Institutes of Health, Bethesda, MD).

### Assessment of the anti-tumour effect in the orthotopic glioblastoma xenograft model

Six-week-old male athymic nude mice (Orientbio) were subjected to a stereotaxic implant of U87MG cells [7 × 10^4^ cells/4 μL in Hank’s buffered saline solution (HBSS)] into the left forebrain at the following coordinates: 2.0 mm lateral and 1 mm anterior to bregma at 3.0 mm depth from the skull surface under anaesthesia (n = 4). Seven days after the tumour cell injection, the mice were intracranially injected with PBS, H5CmTERT-Ad, or H5CmTERT-Ad/TRAIL (5 × 10^9^ VPs) at a constant pumping rate of 0.8 μL/min. At 17 days after the cell injection, all the animals were euthanised. To evaluate tumour by histology and immunohistochemistry, the brains were removed, placed in a 4% paraformaldehyde solution, embedded in paraffin and cut into 5-μm sections (Wax-it, Vancouver, BC, Canada). Representative slices were stained with H & E and then examined by light microscopy. The same paraffin slides were also processed for detection of TRAIL- and TUNEL-positive spots as described above. Tumour slides were also stained with a rabbit anti-E1A IgG Ab (Santa Cruz Biotechnology) and then treated with an ABC-peroxidase kit (ChemoMate DAKO Envision kit; DAKO) to assess viral replication in tumour tissue. The expression levels of TRAIL-, E1A-, and TUNEL-positive cells were semi-quantitatively analysed by means of the MetaMorph image analysis software (Universal Image).

### Assessment of the anti-tumour effect in the U87MG/Fluc orthotopic glioblastoma xenograft model

U87MG/Fluc orthotopic glioblastoma tumour model was prepared as described above using firefly luciferase-expressing U87MG cells for the evaluation of anti-tumour effect (n = 6). Seven days after the tumour cell injection, the mice were intracranially injected with PBS, H5CmTERT-Ad, or H5CmTERT-Ad/TRAIL (5 × 10^9^ VPs) at a constant pumping rate of 0.8 μL/min. Tumour growth was measured every 7 days after the first treatment by bioluminescence imaging using the IVIS imaging system (Xenogen, Alameda, CA). *In vivo* bioluminescence signals were calculated as the sum of the both prone and supine acquisitions for each mouse after background subtraction of total flux [photons/s (p/s)] from a total body region of interest. Cancer progression was monitored until a predetermined endpoint based on overall health. Survival curve was then plotted against time after treatment. To evaluate tumour tissue by histology and immunohistochemistry, the brains were removed, placed in a 4% paraformaldehyde solution, embedded in paraffin and cut into 5-μm sections (Wax-it) at 10 days after the cell injection. Representative slices were stained with H & E and then examined by light microscopy. The same paraffin slides were also processed for detection of TRAIL- and TUNEL-positive spots as described above. Tumour slides were also stained with a rabbit anti-E1A IgG Ab (Santa Cruz Biotechnology) and then treated with an ABC-peroxidase kit (ChemoMate DAKO Envision kit; DAKO) to assess viral replication in tumour tissues.

### Statistical analysis

Data were expressed as mean ± standard deviation (SD). Statistical significance was determined by two-tailed Student’s T test (SPSS 13.0 software; SPSS, Chicago, IL). Data with *P* values less than 0.05 were considered statistically significant.

## Electronic supplementary material

Supplementary Figures
