# Supplementary material for: A hypoxia- and telomerase-responsive oncolytic adenovirus expressing secretable trimeric TRAIL triggers tumour-specific apoptosis and promotes viral dispersion in TRAIL-resistant glioblastoma
Source: Sci Rep. 2018 Jan 23;8:1420. doi: 10.1038/s41598-018-19300-6 (PMC5780382; doi:10.1038/s41598-018-19300-6)
Supplement: Supplementary file 1 — Supplementary Figures [file 41598_2018_19300_MOESM1_ESM.pdf]

A hypoxia- and telomerase-responsive oncolytic adenovirus expressing secretable trimeric TRAIL triggers tumour-specific apoptosis and promotes viral dispersion in TRAIL-resistant glioblastoma

Eonju Oh[1], JinWoo Hong[1], Oh-Joon Kwon[2], and Chae-Ok Yun[1]*

[1]Department of Bioengineering, College of Engineering, Hanyang University, 222 Wangsimni-ro, Seongdong-gu, Seoul 133-791, Korea.

[2]Department of Molecular and Cellular Biology, Baylor College of Medicine, Houston, TX 77030, USA

Running Title: Hypoxia- and telomerase-responsive oncolytic adenovirus expressing stTRAIL

Keywords: hypoxia-responsive, cancer-specific promoter, oncolytic adenovirus, stTRAIL, apoptosis, TRAIL resistance, glioblastoma

---

*Correspondence should be addressed to C.-O. Yun (chaeok@hanyang.ac.kr), Department of Bioengineering, College of Engineering, Hanyang University, 222 Wangsimni-ro, Seongdong-gu, Seoul 04763, Korea

Tel.: +82 2-2220-0491; Fax: +82 2-2220-4850

Supporting Information

Materials and Methods

Comparative transcriptional activity of cancer cell-specific promoter

To assess the transcriptional activity of the mTERT, HmTERT, 5CmTERT, or H5CmTERT promoters under normoxic and hypoxic conditions, glioblastoma cells (U87MG and U251N) were transfected with green fluorescent protein (GFP)-expressing plasmids under the control of the mTERT, HmTERT, 5CmTERT, or H5CmTERT promoters. At 48 h post-transfection, the GFP expression levels were quantified using a microplate reader.

Supplementary Figure 1

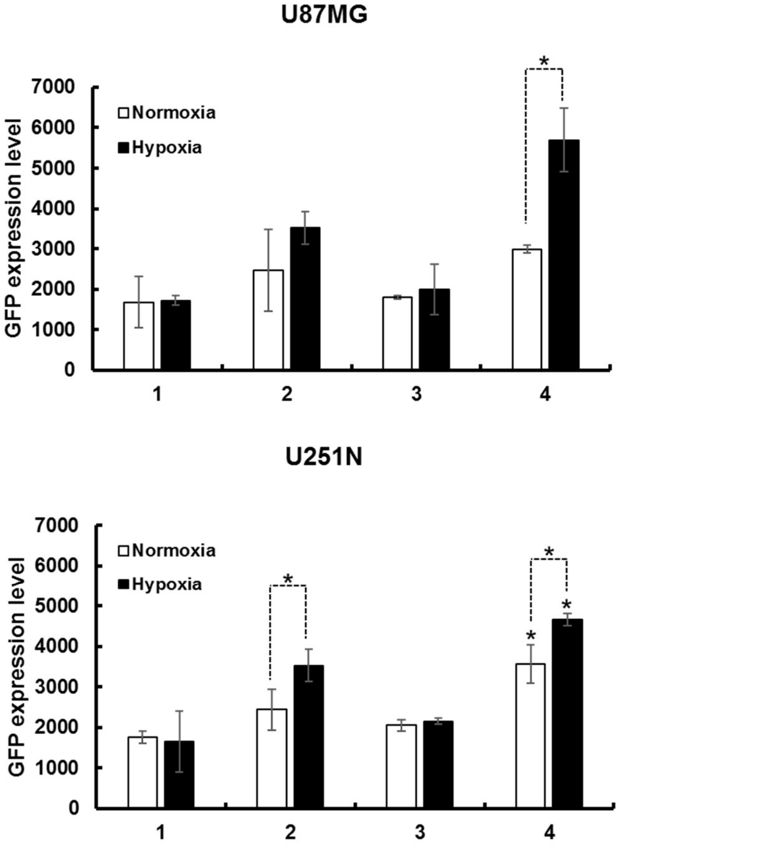

1. mTERT-GFP 2. HmTERT-GFP 3. 5CmTERT-GFP 4. H5CmTERT-GFP

Supplementary Figure 1. GFP expression driven by the cancer cell-specific promoter. The GFP expression level driven by the mTERT, HmTERT, 5CmTERT, or H5CmTERT promoter in glioblastoma cells (U87MG and U251N). GBM cells were transfected with plasmids expressing GFP under the control of the mTERT, HmTERT, 5CmTERT, or H5CmTERT promoter (mTERT-GFP, HmTERT-GFP, 5CmTERT-GFP, or H5CmTERT-GFP) under normoxic or hypoxic condition. GFP expression levels from each group were analysed using a microplate reader after 48 h incubation at 37°C. Each cell line was tested at least three times and data are shown as mean ± SD of triplicate experiments; **P* < 0.05 versus HmTERT-GFP for U251N.

Supplementary Figure 2

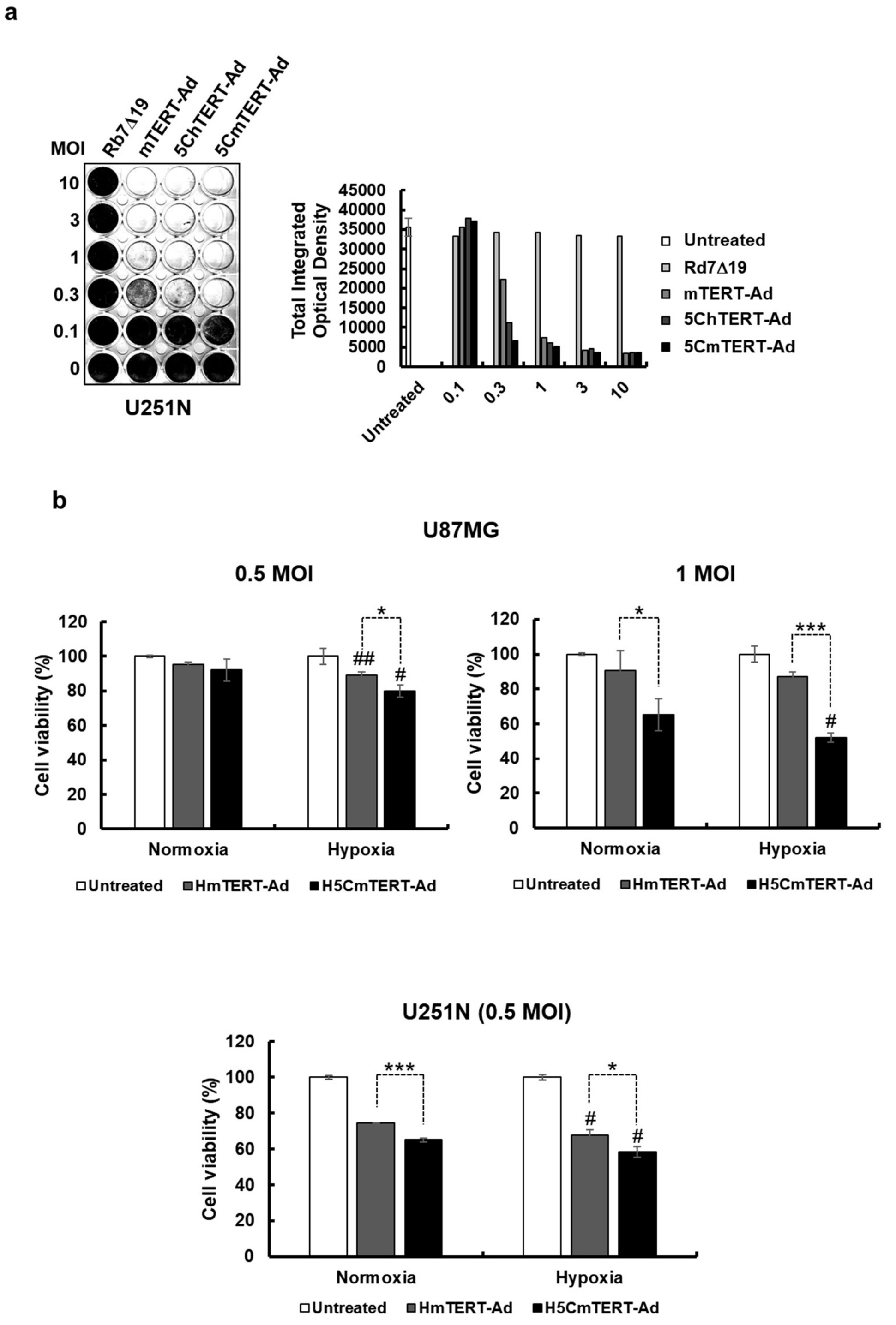

Supplementary Figure 2. Cytopathic effect of the oncolytic adenoviruses. (a) The cytopathic effect of oncolytic adenoviruses (Rb7Δ19, mTERT-Ad, 5ChTERT-Ad, or 5CmTERT-Ad) in U251N cells. At 72 h after the infection, virus-mediated attenuation in cell viability was assessed by the cytopathic effect assay. The result of cytopathic effect assay was semi-quantitatively assessed using ImageJ software. (b) Glioblastoma cells (U87MG and U251N) were infected with HmTERT-Ad or H5CmTERT-Ad at the indicated MOI and incubated under normoxic or hypoxic condition. At 48 h after the infection, the cell viability was determined by the MTT assay. Each cell line was tested at least 3 times and data are shown as mean ± SD of triplicate experiments; *$P < 0.05$, ***$P < 0.001$. $^{\#}P < 0.05$, $^{\#\#}P < 0.01$; cell killing effect under normoxia vs. hypoxia of same virus.

Supplementary Figure 3

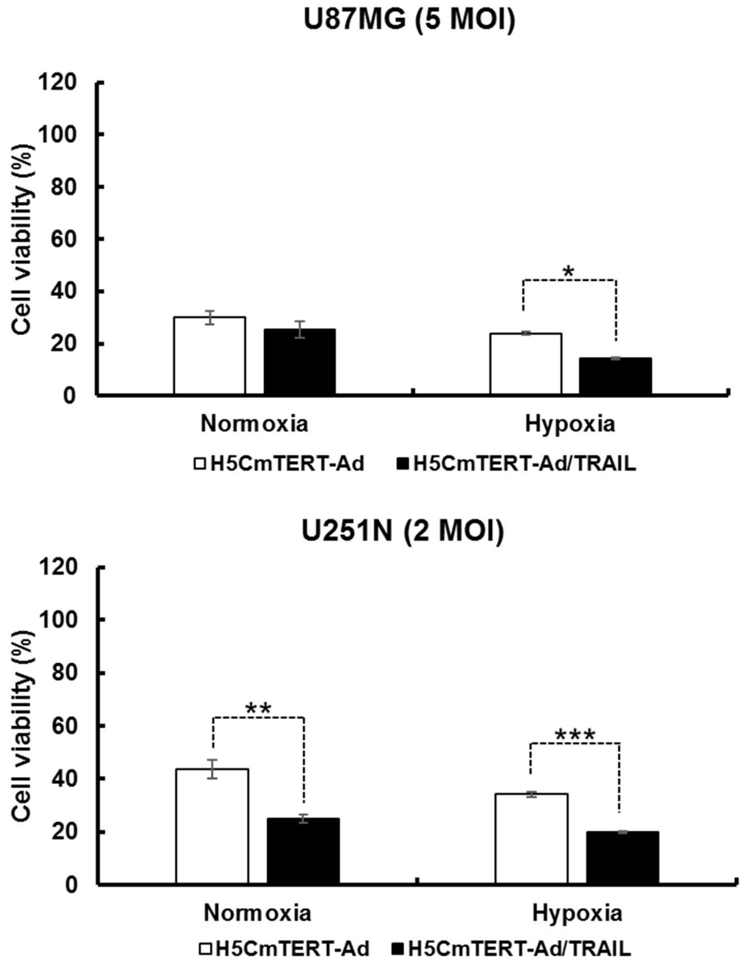

Supplementary Figure 3. Cancer cell killing effect of the oncolytic adenoviruses. Glioblastoma cells (U87MG and U251N) were infected with H5CmTERT-Ad or H5CmTERT-Ad/TRAIL at the indicated MOI and incubated under normoxic or hypoxic condition. At 48 h after the infection, the cell viability was assessed by the MTT assay. Each cell line was tested at least three times and data are shown as mean ± SD of triplicate experiments.

Supplementary Figure 4

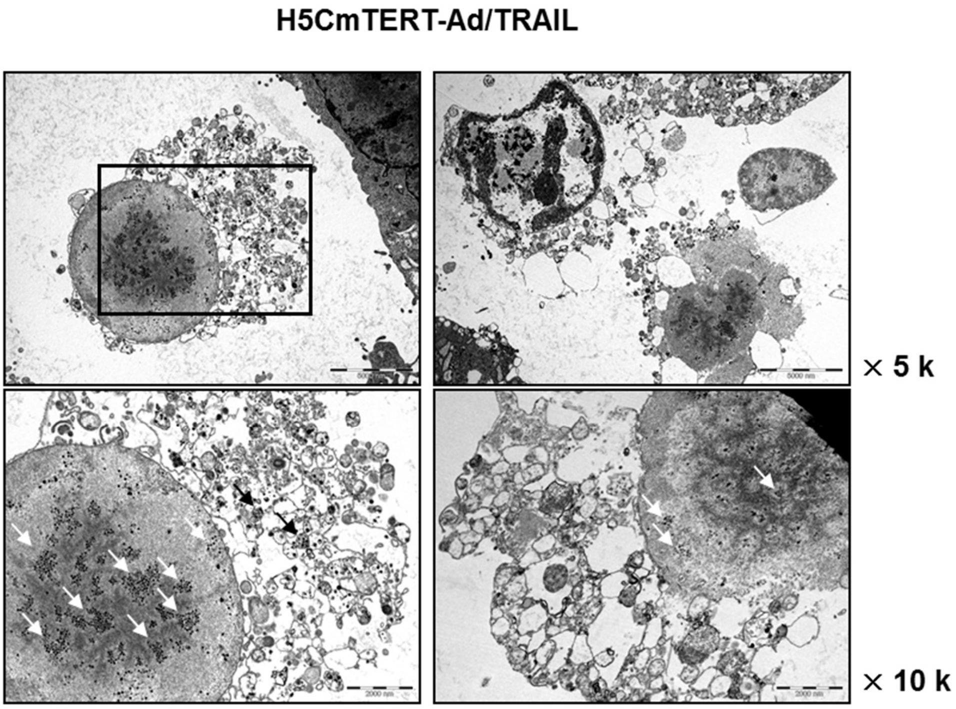

Supplementary Figure 4. Transmission electron microscopy images of U87MG cells after infection with H5CmTERT-Ad/TRAIL. U87MG cells were treated with H5CmTERT-Ad/TRAIL. At 36 h post-infection, the cells were harvested and analysed by transmission electron microscopy. Arrows indicate multiple adenovirus particles in U87MG cells. Original magnification: ×5,000 and ×10,000.

Supplementary Figure 5

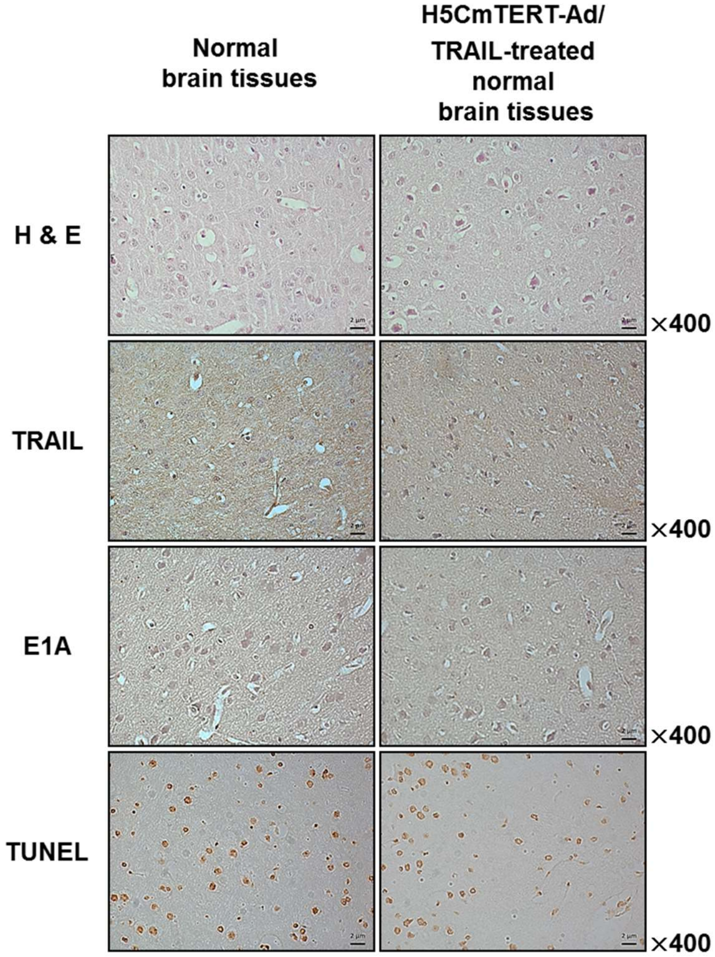

Supplementary Figure 5. Histological and immunohistochemical analyses of normal brain tissues treated with H5CmTERT-Ad/TRAIL. H5CmTERT-Ad/TRAIL (5×10^9 VPs) was administered to normal brain tissue of mice via intracranial injection. At 3 days after the viral injection, the mice were euthanised and the brains were collected. Representative sections were stained with H & E. The expression levels of TRAIL and E1A were assessed by immunohistochemical analysis. A TUNEL assay was performed to detect apoptosis. Data are representative of three independent experiments. Original magnification: ×400.

Supplementary Figure 6

a

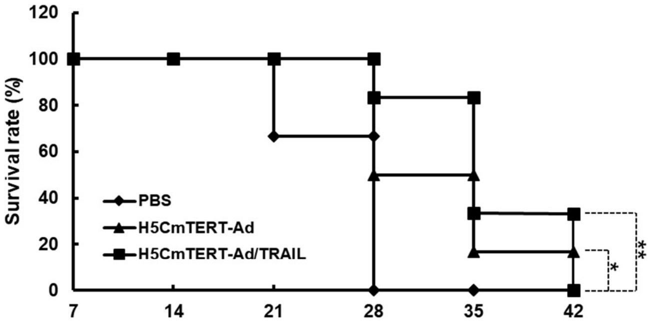

Group	The percentage of surviving mice (At 35 days after cell injection)	Survival rate *P*-value (vs. PBS)
PBS	0%	-
H5CmTERT-Ad	16.7%	$P < 0.05$
H5CmTERT-Ad/TRAIL	33.3%	$P < 0.01$

b

Group	Actual median survival time after cell injection (days)	Actual median survival *P*-value (vs. PBS or H5CmTERT-Ad)
PBS	30	-
H5CmTERT-Ad	30	-
H5CmTERT-Ad/TRAIL	> 35	$P < 0.05$

Supplementary Figure 6. Survival curve analysis of the U87MG/Fluc orthotopic glioblastoma tumour model. (a) Orthotopic glioblastoma tumour model was established by stereotaxically implanting firefly luciferase-expressing U87MG (U87MG/Fluc) cells into the left forebrain of nude mice. At 7 days after the tumour cell injection, PBS, H5CmTERT-Rd19-Ad, or H5CmTERT-Ad/TRAIL (5 × 10^9 VPs) were administered via intracranial injection. The percentage of surviving mice was determined by monitoring the tumour growth-related events

(mice were considered dead when total flux exceeded 1×10^8 p/s). Data presented as mean ± SD; *P < 0.05, **P < 0.01. (b) Median survival time represents the day at which 50% mortality is actually observed in the treatment group. Data presented as mean ± SD; *P < 0.05; H5CmTERT-Ad/TRAIL vs. PBS or H5CmTERT-Ad.
